# Immune Responses and Protective Efficacy of a Formalin-Killed *Francisella Noatunensis* Subsp. *Orientalis* Vaccine Evaluated through Intraperitoneal and Immersion Challenge Methods in *Oreochromis Niloticus*

**DOI:** 10.3390/vaccines8020163

**Published:** 2020-04-03

**Authors:** Theeraporn Pulpipat, Shun Maekawa, Pei-Chi Wang, Shih-Chu Chen

**Affiliations:** 1 Department of Veterinary Medicine, College of Veterinary Medicine, National Pingtung University of Science and Technology, Pingtung 91201, Taiwan; pidpudpan@gmail.com (T.P.); shun84topaz04@gmail.com (S.M.); pc921003@gmail.com (P.-C.W.); 2 International Degree Program of Ornamental Fish Technology and Aquatic Animal Health, International College, National Pingtung University of Science and Technology, Pingtung 91201, Taiwan; 3 Research Center for Fish Vaccine and Diseases, College of Veterinary Medicine, National Pingtung University of Science and Technology, Pingtung 91201, Taiwan; 4 Southern Taiwan Fish Diseases Research Center, College of Veterinary Medicine, National Pingtung University of Science and Technology, Pingtung 91201, Taiwan; 5 Research Center for Animal Biologics, National Pingtung University of Science and Technology, Pingtung 91201, Taiwan

**Keywords:** *Francisella noatunensis* susbsp. *orientalis*, formalin-killed vaccine, *Oreochromis niloticus*, immune response

## Abstract

*Francisella noatunensis* subsp. *orientalis* (*Fno*), an intracellular bacterium, causes systemic granulomatous diseases, resulting in high mortality and huge economic losses in Taiwanese tilapia farming. In this study, we tested the efficacy of a formalin-killed *Fno* vaccine in cultured tilapia. *Fno* was isolated from diseased tilapia, inactivated with formalin, and mixed with the mineral oil base adjuvant (Montanide^TM^ ISA 763 AVG). A total of 300 tilapia were divided into two groups. The experimental group was intraperitoneally injected with 0.1 mL of vaccine, which was substituted with phosphate-buffered saline (PBS) in the control group. A booster was administered at 2 weeks post-immunization. Tilapia were challenged at 6 weeks post primary immunization by intraperitoneal (IP) injection and immersion methods. Mortality was recorded at 21 and 60 days. The results revealed that the vaccine induced a greater antibody titer and led to 71% and 76% of relative percent survival (RPS) after the IP and immersion challenge. The transcripts of proinflammatory cytokines and immune-related genes, including interleukin-1β (IL-1β), tumor necrosis factor alpha (TNFα), C-X-C motif chemokine ligand 8 (CXCL8), and interleukin-17C (IL-17C), were significantly upregulated after vaccination. Additionally, vaccinated fish had lower bacterial loads in the blood and lower granuloma intensities in the kidney, spleen, liver, and gill than control fish. The results in this study demonstrate that the inactivated *Fno* vaccine could be an essential resource in Taiwanese tilapia farming.

## 1. Introduction

*Francisella noatunensis* subsp. *orientalis*, also known as *F. asiatica*, is a Gram-negative intracellular pathogenic bacterium that has a coccobacillus shape and fastidious nature. It causes acute or chronic systemic granulomatous disease in both freshwater and seawater fish [1,2]. Tilapia is the major species affected by *Fno*. The disease causes high morbidity, with mortalities ranging from 20% to 95%, resulting in significant economic losses in tilapia farming worldwide [1,2,3]. Tilapia francisellosis was initially reported in Taiwan in 2005 as a *Francisella*-like bacterium in cultured tilapia [4] and is still considered a major threat in Taiwanese tilapia farming [5] along with other bacterial diseases like streptococcosis [6,7]. However, streptococcosis usually occurs in the summer season, while *Fno* generally causes disease outbreaks in the winter season. Nevertheless, both of the two diseases have a huge economic impact on the tilapia farming industry worldwide. In a recent study, our group reported that the phenotypic characteristics and enzymatic profiles using API ZYM kits (bioMérieux, Marcy l’Etoile, France) of the Taiwanese *Fno* strains were identical between tilapia and the Green Texas cichlid (*Herichthys cyanoguttatus*). In addition, there was a high degree of genetic homogeneity (16S rRNA, housekeeping genes sequencing, and pulsed-field gel electrophoresis (PFGE) typing) between the *Fno* strains isolated from the two species of fish. However, the phenotypic characteristics of Taiwanese *Fno* strains differed slightly from the *Fno* Ehime-1 strain isolated from three-line grunt (*Parapristipoma trilineatum*) in Japan [8] and the *Fno* STIR-GUS-F2f7 strain from red Nile tilapia (*O. niloticus*) in the United Kingdom [9].

Even though antibiotics are generally a good choice for managing bacterial infections in fish farming, francisellosis is difficult to treat with antibiotics [1,2] because *Fno* is an intracellular bacterium that is primarily found inside cytoplasmic vacuoles within macrophages [10,11]. Moreover, the emergence of antibiotic-resistant bacterial strains is a severe consequence of the overuse or misuse of antibiotics. Therefore, vaccination is a more effective strategy for controlling and preventing tilapia francisellosis [12,13,14,15]. Currently, approved commercial vaccines against tilapia francisellosis are not available. The first successful vaccination attempt against *Fno* was reported in 2010 with the use of a genetically-modified attenuated *Fno* vaccine (Δ*iglC*) [13]. However, concrete and extensive evidence of the safe use of a live attenuated vaccine is urgently needed. The utilization of Genetically Modified Organism (GMO)-based vaccination for fish followed by their release into open water fish farms is highly limited and is still not allowed in aquaculture worldwide [14,16,17]. An inactivated vaccine would overcome this problem and would also be economically feasible for use in a cheap fish like tilapia. The key strategy in developing an effective inactivated vaccine is identifying suitable antigens/strains that can induce protective immune responses against the pathogen and that are safe for the environment. Two recent studies investigated the effect of an inactivated vaccine on tilapia francisellosis. A whole cell formalin-inactivated vaccine was first developed using the highly virulent isolate STIR-GUS-F2f7 and the oil-based adjuvant Montanide™ ISA 763 AVG [14]. This vaccine elicited good protective efficacy (relative percent survival (RPS) 100%) against francisellosis in red Nile tilapia. Shahin et al. [12] also evaluated the efficacy of this vaccine against two heterologous *Fno* strains (from different geographical regions) in Nile tilapia. The results revealed that the vaccinated fish challenged with the homologous isolate showed high protection (an RPS of 82.3%), whereas fish challenged with the two heterologous isolates showed RPSs of 69.8% and 65.9%. These differences in efficacy could be the result of the genotypic and phenotypic differences between the vaccine candidate strains and the challenge strains. The homologous vaccine strain was able to provide a high protection against challenge with the same strain but the cross protection against challenge with the heterologous strains was not as high. The Taiwanese isolates showed phenotypic variation when compared to other isolates from different geographical regions. Therefore, there could be a similar situation in Taiwan. Presently, there have been no studies demonstrating vaccine efficacy against tilapia francisellosis using local strains. That is why further studies are needed to develop an efficacious vaccine based on local strains.

This study was a pilot investigation of a vaccine against tilapia francisellosis in Taiwan. We developed an injectable formalin-killed *Fno* vaccine based on local highly virulent bacterial strains isolated from Taiwanese cultured tilapia. We evaluated the efficacy of the vaccine via two different challenge methods (intraperitoneal injection and immersion method). The immersion challenge route was opted for due to its similarity in natural bacterial infection. The specific antibody titer, the expression profiles of immune-related genes after vaccination, bacterial invasion and clearance, and the granuloma score in vaccinated fish after challenge with *Fno* were evaluated.

## 2. Materials and Methods

### 2.1. Fish and Rearing Management

Healthy, francisellosis-free Nile tilapia (*Oreochromis niloticus*) (35 ± 5 g) obtained from a fish farm in Taiwan were used in this study. Tilapia were reared in 2000 L tanks with a controlled water temperature of 28 ± 1 °C in aerated fresh water. Fish were fed with commercial dry pellets corresponding to 3% of their total body weight and acclimatized for 2 weeks before performing the experiments. The protocols used in this study were completed according to guidelines of the Animal Use Protocol and the Institutional Animal Care and Use Committee of the National Pingtung University of Science and Technology, Taiwan under protocol number NPUST-107-028, 2018.

### 2.2. Bacteria Strain and Vaccine Preparation

The *Fno* AOD104086 strain was originally isolated from cultured tilapia in Taiwan, and has been described in a previous study [5]. The *Fno* strain was recovered from the 20% skim milk stock solution and cultured on cysteine heart agar supplemented with 2% hemoglobin (CHAH) (BD BBL, Sparks, MD, USA) at 25 °C for 72 h. A single colony was sub-cultured in modified brain heart infusion broth (BD BBL, Sparks, MD, USA) at 25 °C with shaking at 150 rpm for 60 h, as previously published [18]. The culture medium was centrifuged at 3500 × g for 20 min, and then the bacterial pellet was washed 3 times with sterile phosphate-buffered saline (PBS). The bacteria were suspended in a solution of 0.3% formalin in PBS (10^10^ colony-forming unit (CFU)/mL)) and slowly shaken for 48 h at 25 °C. Inactivation of the bacteria was confirmed by inoculating on to CHAH agar and incubating at 25 °C for 72 h. The completely inactivated bacterial cells were washed 3 times with sterile PBS to remove formalin. Commercial adjuvant Montanide^TM^ ISA 763 AVG (SEPPIC, Paris, France) was emulsified with the inactivated bacteria at a volume ratio of 30% antigen to 70% adjuvant (10^9^ CFU/mL final concentration).

### 2.3. Vaccine Safety and Fish Immunization

#### 2.3.1. Vaccine Safety Test

To test the safety of the vaccine, ten fish were intraperitoneally (IP) injected with 0.2 mL of the vaccine (two times the vaccination dose). No adverse side effects were observed in the injected fish. Observed adverse effects included behavior changes (lethargy, appetite, aggression, gaping, flashing, and color changes), mortalities, toxicity, or other clinical signs related to IP injection at a high dose for 14 days.

#### 2.3.2. Immunization and Sample Collection

Tilapia were randomly divided into two groups (vaccine and control groups), and each group had 150 fish. Tilapia were anesthetized using tricaine methanesulfonate (MS222). Fish were IP injected with 0.1 mL of the vaccine (10^8^ CFU/fish) or the same volume of PBS as the control group. At 14 days after the first immunization, booster immunizations were conducted in the vaccinated groups. Randomly, the spleens and kidneys from 6 fish of the control and vaccinated groups were collected at 24, 48, and 72 h after the primary immunization for immune related gene analysis by reverse transcriptase real-time PCR (RT-qPCR). Furthermore, serum was collected from 10 randomly selected fish per group at week 2 and week 6 after primary immunization. The serum was kept in −80 °C for the measurement of the antibody titer by the enzyme-linked immunosorbent assay (ELISA).

#### 2.3.3. Specific IgM Antibody Titer Analysis by ELISA

The specific serum antibody concentrations after vaccination were determined based on an indirect ELISA method as previously described, with some modifications [19]. Firstly, Nunc-Immuno 96 MicroWell solid plates, (Sigma, St. Louis, MO, USA) were coated with 100 µL *Fno* sonicated antigen (approximately 2 × l0^7^ CFU/well) and the plates were then incubated overnight at 4 °C. The antigen-coated plates were blocked with 2% skim milk at room temperature for 2 h to prevent nonspecific immune reactions. The plates were washed 3 times with PBS containing 0.03% Tween 20 (PBST). Antigen-coated plates were made to subsequently react with antiserum from vaccinated or control fish. The antisera from fish were diluted with PBS at a ratio of 1:250. The specific IgM antibody was bound with rabbit antiserum against tilapia IgM (diluted 1:1000 in PBST). Each sample was added in duplicate, and negative control serum was introduced to each plate. Negative control sera from tilapia were collected from non-vaccinated fish. Following the incubation of goat antiserum against rabbit IgG conjugated with horseradish peroxidase (Santa Cruz Biotechnology, Dallas, TX, USA) (diluted 1:5000 in PBST) for 1 h at 25 °C, wells were washed with PBST 5 times. Then, the wells were subjected to incubation with a substrate buffer (0.6 mg/mL of 2,20 -amino-bis 3-ethylbenzothiazoline-6-sulfonic acid diammonium salt in 0.1 M citric acid buffer; pH 4.0) containing 0.003% H_2_O_2_ for 15 min at 25 °C, which resulted in a color reaction that was promptly stopped with the addition of 1% sodium dodecyl sulfate. Each plate was interpreted using a Multiskan Spectrum Microplate Spectrophotometer (Thermo Fisher Scientific, Vantaa, Finland) at the 450 nm wavelength.

#### 2.3.4. Immune Related Gene Analysis

Total RNA was isolated from the spleens and head kidneys of fish from all groups using TRIzol^®^ reagent (Invitrogen Corp., Carlsbad, CA, USA). The RNA was then quantified, and the quality was checked based on absorbance at 260 nm by the Nanophotometer (Implen, München, Germany). Single-strand cDNA was synthesized from 1 µg total RNA using an iScript™ cDNA Synthesis Kit (Bio-Rad Laboratories Inc., Hercules, CA, USA). The mRNA expression levels of interleukin-1β (IL-1β), tumor necrosis factor alpha (TNFα), C-X-C motif chemokine ligand 8 (CXCL8) or interleukin-8 (IL-8), and interleukin-17C (IL-17C) were determined by using specific primers as listed in Table 1. 

All specific primers used in this study were designed based on the available nucleotide sequences from the NCBI GenBank database. qRT-PCR was performed in 96-well plates using the iQ™ SYBR Green Supermix (Bio-Rad Laboratories Inc., Hercules, CA, USA). according to manufacturer’s instructions. Individual 10 µL reactions consisted of 5 µL iQ™ SYBR Green Supermix), 300 nM primers, and cDNA diluted at 1:20 as the template. The optimal annealing temperature for all primers was determined using the thermal gradient feature of the CFX96 Real-time PCR detection system (Bio-Rad Laboratories Inc., Hercules, CA, USA). Product amplification was detected using SYBR Green fluorescence during the 56 °C step. The ribosomal protein L23 (RPL23) served as an internal control for cDNA normalization. Gene expression was calculated as relative to the RPL23 gene using the 2−ΔΔCt method [20].

#### 2.3.5. Intraperitoneal and Immersion Challenge Tests

To evaluate the resistance of vaccinated fish to the *Fno* vaccine strain, at six weeks post primary immunization, control and vaccinated fish were challenged by the IP route and immersion method with a dose of 10 LD_50_ (median lethal dose) in each group (n = 30 with duplications). The LD_50_ dose used in the IP injection was approximately 10^4^ CFU/fish, similar to that in a previous study [5], whereas the LD_50_ dose used in the immersion method was around 10^7^ CFU/mL of water as described in a previously published protocol [21]. Before challenge, all fish were starved for 24 h and anesthetized with MS222 (100 mg/L). The fish were IP injected with 0.1 mL of bacterial suspension with a concentration of 10 LD_50_ (1.75 × 10^5^ CFU/fish). For the immersion challenge, fish were immersed in 10 L of static water containing a bacterial dose of 10 LD_50_ (1.75 ×10^8^ CFU/mL of water) for 3 h and then moved to a new tank. All of the fish in the experimental challenge were raised in the tank with a circulation system, and the water was maintained at 25 ± 2 °C to mimic the natural outbreak condition. Mortality was observed and recorded for 21 days after the IP challenge and 60 days after the immersion challenge. The protection efficacy of the vaccine was determined by comparing the cumulative mortalities of the vaccine and control groups. The RPS was calculated according to the equation:

(1) $$\mathrm{RPS}=\left(1-\left(\frac{\%\;\mathrm{mortality}\;\mathrm{of}\;\mathrm{vaccine}\;\mathrm{group}}{\%\;\mathrm{mortality}\;\mathrm{of}\;\mathrm{control}\;\mathrm{group}}\right)\right)\times 100$$

#### 2.3.6. Blood Bacterial Invasion and Clearance after Challenge

The bacterial concentrations in the blood of the challenged tilapia were determined at 0, 24, 48, 72, 120, and 168 h post-challenge, by measuring the numbers of colony forming units per milliliter of blood, following a previously published protocol [22]. Blood (0.1 mL) from the caudal vein was aseptically collected from three individual tilapia in the control and vaccine groups. Each blood sample was mixed with 0.9 mL of sterile PBS and homogenized by thoroughly vortexing the solution. A 10-fold serial dilution of each sample was used to assess plate counts. Each dilution was spread over CHAH agar plates and incubated at 25 °C for 96 h. The CFU/mL was then calculated.

#### 2.3.7. Granuloma Score

During the observation period for challenge, all dead and surviving fish in each group were visually inspected for granulomas in the spleen, kidney, liver, and gills. The level of intensity of the granulomas or white nodules was categorized, based on a previously published protocol, as follows [23]: level: (**-**) no white nodules, (+) 1–5 nodules, (++) 6–10 nodules, and (+++) more than 10 nodules. The intensities of the granuloma scores were calculated as the percentages of occurrence among 30 fish per group.

#### 2.3.8. Statistical Analysis

GraphPad PRISM software version 8.0 (GraphPad Software, Inc., La Jolla, CA, USA) was used for statistical analysis and the creation of graphs. Student’s t tests were used for the statistical analysis of the differences in the serum antibody titers, immune related gene expression profiles, and blood bacterial concentrations after the *Fno* challenge, between control and vaccinated fish at all time points. Statistical significance was determined using the Holm-Sidak method, with alpha = 0.05 and denoted as *p* < 0.05 (*), *p* < 0.01 (**), or *p* < 0.001(***) in figures. The blood bacterial concentration after the *Fno* challenge between control and vaccinated fish at all time points was also analyzed by a one-way analysis of variance (ANOVA) and Tukey’s post hoc test.

## 3. Results

### 3.1. Safety of the Vaccine

All ten fish injected with 0.2 mL of formalin-inactivated Fno vaccine were healthy and exhibited normal behavior. The necropsy findings of these tilapia showed no adverse impacts such as adhesion and melanization, or any inflammatory lesions in the abdominal cavity. White vaccine droplets were observed in the abdomen.

### 3.2. Specific IgM Antibody Titers

**Figure 1:**
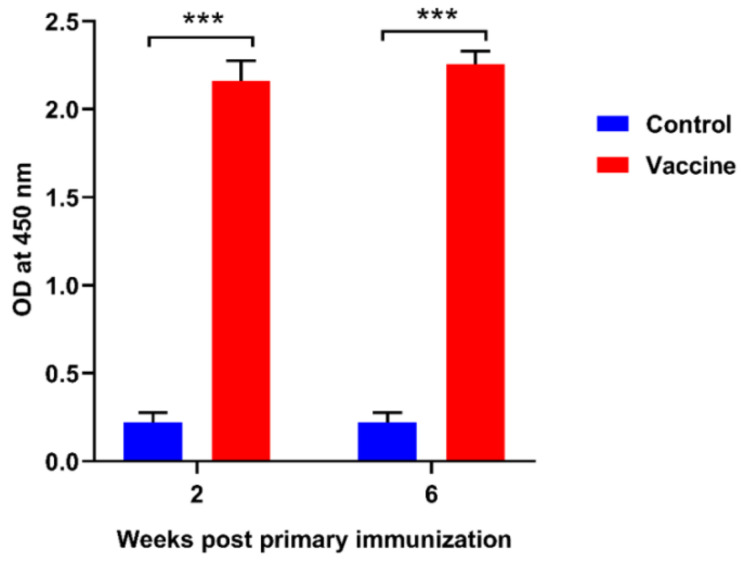
Specific IgM antibody titers were determined by the ELISA assay from 10 fish at 2 weeks and 6 weeks after primary immunization. Asterisks (***) indicate significant differences, with *p* < 0.001, from the control at each time point.

The specific IgM antibody titer in vaccinated fish was significantly higher than in control fish (*p* < 0.001) (Figure 1). The antibody titer in the vaccinated fish gradually increased at weeks 2 and 6 after the first immunization. The specific IgM antibody titer was high and consistent with the RPS result.

### 3.3. Immune Related Gene Analysis after Immunization

**Figure 2:**
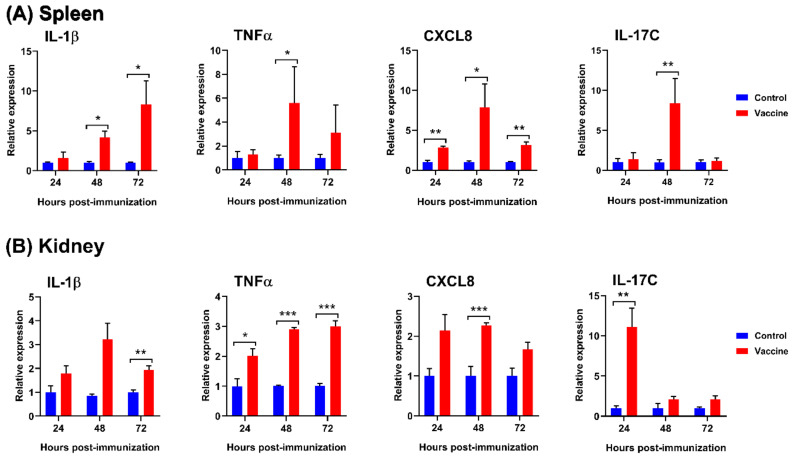
Comparative expression profiles of IL-1β, TNFα, CXCL8, and IL-17C in the spleen (**A**) and kidney (**B**) from control and vaccinated fish (n = 6) at 24, 48, and 72 h after primary immunization. The expression of target genes was normalized to that of the RPL23 gene as a reference gene. The graph shows the relative expression levels in the vaccinated and control groups. Asterisks (*), (**), and (***) indicate significant differences with *p* < 0.05, *p* < 0.01, and *p* < 0.001, respectively, from the control at each time point.

RT-qPCR was performed to investigate the effect of vaccination on the expression of immune-related genes encoding IL-1β, TNFα, CXCL8, and IL-17C in the spleen and head kidney at 24, 48, and 72 h after immunization. The results showed that all examined genes were significantly upregulated in vaccinated fish, compared to in the control groups (Figure 2). IL-1β gene expression was gradually upregulated in the spleen from 24 h and peaked at 72 h post-immunization. In the kidney, the expression of IL-1β markedly increased at 24 to 48 h post-immunization and slightly decreased thereafter, but was still higher than in the control group at 72 h post-immunization. TNFα gene expression in the spleen was upregulated to the highest level at 48 h post-immunization, but gradually decreased at 72 h post-immunization. There was a stable upregulation of TNFα gene expression in the kidney from 24 h to 48 h, which peaked at 72 h post-immunization. CXCL8 gene expression increased in the spleen and kidney, peaking at 48 h post-immunization, and then decreased at 72 h post-immunization. IL-17C gene expression in the spleen was not upregulated at 24 h post-immunization, but was rapidly upregulated to a peak at 48 h and later downregulated at 72 h post-immunization. However, IL-17C expression was rapidly increased in the kidney at 24 h post-immunization, with a stable decrease at 48 h and 72 h post-immunization.

### 3.4. Protection of Fish after Immunization (RPS)

**Figure 3:**
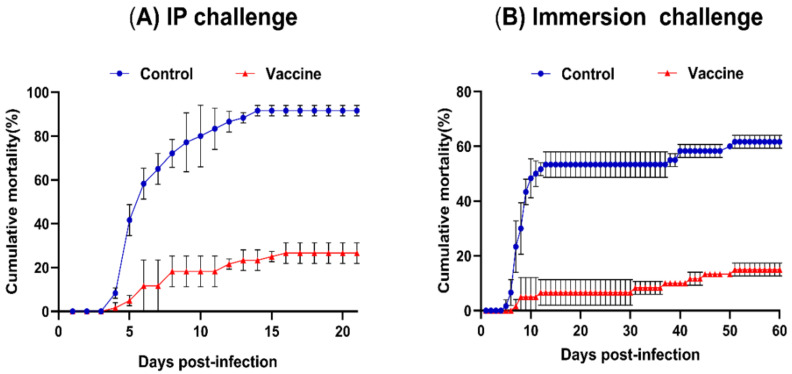
The averages of the cumulative mortalities of tilapia challenged with the Fno vaccine strain via the intraperitoneal (IP) injection method (**A**) and immersion method (**B**) at 6 weeks post primary immunization.

Two comparative challenge experiments (IP and immersion methods) were conducted to evaluate the protective effect of the vaccine. As shown in Figure 3 and Table 2 at 21 and 60 days after the IP and immersion challenges, the vaccinated fish demonstrated significantly lower mortalities (average 26.65% and 15%) than the control groups, under laboratory conditions. The average cumulative mortality reached more than 60% in the control group (IP method, 91.65% and immersion method, 61.67%). The mortality patterns based on the two challenge methods were slightly different. In the IP challenge experiment, mortalities were observed from day 4 to day 14 post-infection, with mortality rates of up to 91.65% and 26.65% in the control and vaccine groups, respectively. No mortality was observed after day 13. However, in the immersion challenge experiment, deaths started in the control group from day 5 post-infection, with the mortality rate reaching 53.33% after 13 days post-challenge, after which no mortality was recorded until day 38. A 8.34% mortality rate was observed between the 38th day and 50th day, bringing the total mortality to 61.67%. However, in the vaccine group, mortality was observed from day 5 and steadily increased until day 50, bringing the total mortality to 15%. Notably, both the IP and immersion methods conferred good protection in the vaccinated groups post-challenge, with RPSs of 71% and 76%, respectively.

### 3.5. Blood Bacterial Invasion and Clearance

**Figure 4:**
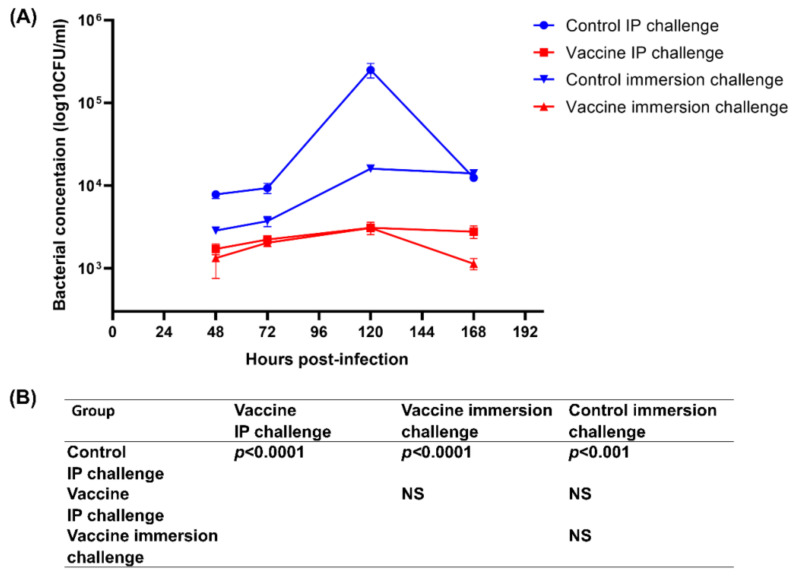
Comparison of blood bacterial concentrations between control and vaccinated fish after 24 h, 48 h, 72 h, 120 h, and 168 h post-infection (n = 3) (**A**). The differences among groups were tested using a one-way ANOVA with Tukey’s post hoc test (**B**). NS = Not significant.

To assess bacteremia as a potential cause of mortality after challenge, peripheral blood from fish in the control and vaccinated groups was subjected to a bacterial count of Fno (Figure 4). The blood bacterial concentration in vaccinated fish was significantly lower than in control fish after challenge by both methods at all time points. For the IP challenge methods, both the control and vaccinated fish presented high bacterial concentrations at 48 h, which gradually increased and peaked at 120 h, and then decreased at 168 h post-infection. The highest bacterial concentration in the blood at 120 h was related to the quick onset of the first dead fish (at day 4 post-infection) and the high mortality rate. With the immersion challenge method, the pattern of blood bacterial invasion was similar to the IP method. In control group of immersion method, we observed highest bacterial number at 120 h post-infection was 15 times lower than the IP method. However, at 168 h post-infection, the bacterial number was consistently higher compared to the IP challenged fish. Additionally, the onset of the first dead fish was also delayed by 1 day.

### 3.6. Granuloma Scores

**Figure 5:**
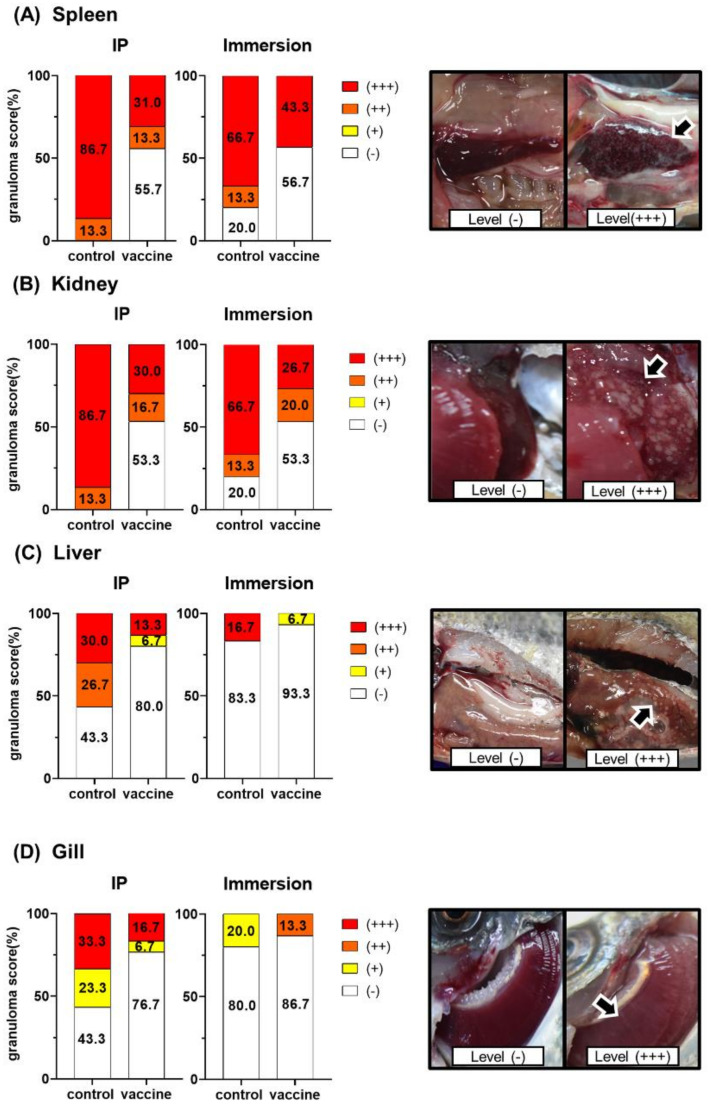
The intensities of granulomas in the spleen (**A**), kidney (**B**), liver (**C**), and gill (**D**) from dead and surviving fish of the control and vaccine groups after the challenge with *Fno* by the IP and immersion methods. The level of intensity of a granuloma or white nodule was categorized as the following: level (-) no white nodules, (+) 1–5 nodules, (++) 6–10 nodules, and (+++) more than 10 nodules [23]. The intensities of the granuloma scores were calculated as percentages of occurrence among 30 fish per group.

All dead and surviving fish in each group demonstrated gross lesions typical of systematic granulomatous disease (whitish nodules), consistent with fish francisellosis, based on the variation in the intensity of the granuloma scores. Macroscopic analyses of the intensities of the granuloma scores in the spleens and head kidneys of the vaccinated fish in the IP challenge group revealed a lower percentage of “level (+++)” than in non-vaccinated fish. All vaccinated fish in both the IP method and the immersion challenge method groups showed “level (-)” in 53.33% of all organs (Figure 5). Additionally, the clinical signs in the acutely infected fish were bloody ascites and slight swellings of the spleen and kidney. However, no granulomas were seen.

## 4. Discussion

Vaccination is an effective and environmentally safe method to control and prevent the outbreak of fish diseases [24]. *Fno* AOD104086 is a local strain which belongs to the clonal group of *Fno* isolates that cause francisellosis in Taiwanese tilapia [5]. Owing to the high virulence of this strain, it was selected as the candidate strain for combination with the oil-based adjuvant Montanide^TM^ ISA 763 AVG for the development of the first vaccine in Taiwan. Several studies have documented that the injectable inactivated formalin-killed vaccines can induce protective antibodies against various bacterial diseases in fish [12,14,19,22,25,26]. The first vaccine for francisellosis was documented by Soto et al., (2011) [13], which was a live attenuated vaccine administered by the immersion method. This kind of vaccine may be feasible in large-scale farms for small fish that are difficult to inject. However, with live vaccines, there is always a risk of pathogens reverting to their virulent state. Therefore, an injectable inactivated vaccine with a commercial adjuvant (the oil-based adjuvant Montanide^TM^ ISA 763 AVG) is a better choice against fish bacterial diseases [12,14,22]. Moreover, the efficacy of immersion vaccines is low or moderate when compared to that of injection vaccines. Many factors should be considered when it comes to the efficacy of immersion vaccines, i.e., the dose of antigen, form of antigen (soluble or particle), uptake of antigen, time of immunization, adjuvant, and fish size or age. Numerous studies have shown that the duration of protection is shorter for immersion vaccines and that the immune response is not efficient either [27,28]. Hence, the present experiment was designed for an injectable inactivated whole-cell vaccine. In this study, the formalin-killed *Fno* vaccine provided greater *Fno*-specific antibody titers than those in non-vaccinated fish at week 2 and week 6 post primary immunization. This indicates that our vaccine successfully triggers the adaptive immunity of vaccinated fish.

Classic proinflammatory cytokine genes including IL-1β and TNFα play a key role in the regulation of the inflammatory process at the early stages of infection in fish, providing the first line of host defense [10,29,30]. The roles of fish IL-1β are the activation of lymphocytes, to function as a chemoattractant for fish leucocytes; leucocyte migration; and the enhancement of the phagocytic and lysozyme activities of macrophages [31]. Additionally, fish IL-1β can also induce the expression of TNFα [32], modulate the expression of the IL-17 family—which is important for antibacterial activity [30], enhance antibody production, and induce the expression of major histocompatibility complex class II β (MHCII β) chain [33]. Fish TNFα reportedly has an overlapping function with IL-1β [30]. The roles of fish TNFα include the regulation of leucocyte proliferation, migration, homing, and the recruitment of phagocytic granulocytes, as well as the induction of cell apoptosis [30,34]. CXCL8 is a chemotactic cytokine that is produced by various cell types to stimulate the recruitment and activation of neutrophils to infection and inflammatory sites [35,36,37,38,39,40,41]. Recent studies in fish have demonstrated that the proinflammatory cytokines IL-1β, TNFα, and CXCL8 are potential adjuvants to promote cytokine immune responses and trigger antibody responses, as well as cellular immune responses, by co-administration with an antigen [42]. Therefore, the transcriptional analysis of proinflammatory cytokine and chemokine gene expression was performed by qRT-PCR in this study. Vaccinated fish demonstrated robust upregulation of proinflammatory cytokine and chemokine genes including, IL-1β, TNFα, and CXCL8 compared to the non-vaccinated fish (Figure 2). These findings correspond with recent studies in tilapia, in which a formalin-killed whole cell *Fno* vaccine enhanced the upregulation of IL-1β and TNFα in the spleen and significantly upregulated IL-1β in the kidney cells of adult zebrafish vaccinated with outer membrane vesicles (OMVs) from *Fno* at 24 h post-immunization [12,15]. Moreover, the observed marked upregulation of IL-1β was also similar to the early inflammatory response to an experimental *Fno* infection in tilapia, in which *Fno* stimulated a significantly higher IL-1β expression in the spleens of Nile tilapia at 24–96 h post-infection [43]. These results demonstrate that our vaccine was capable of activating an early immune response and stimulating an inflammatory process in fish, which increased the host’s ability to eliminate the pathogen [44]. Furthermore, a significant up-regulation of IL-17C was also observed in the spleen and head kidney of vaccinated fish (Figure 2). IL-17 has been identified in vertebrates as a key player in innate immune responses for protection against pathogens [45]. These data also appear to be consistent with other research showing the upregulation of IL-17C in trout infected with *Yersinia ruckeri* [46].

In this study, we evaluated two different challenge methods—specifically, the IP injection and immersion methods—in order to confirm vaccine efficacy. Traditionally, the IP injection challenge method is the most common for evaluating vaccine efficacy because it is a reliable and reproducible challenge model [24]. However, this direct injection method does not accurately mimic natural infection [16] even though it guarantees an equal challenge dose in all fish [24]. The immersion challenge method mimics waterborne natural infection by cohabitation; however, the challenge dose per fish is unequal [47]. Therefore, our results include some variability in disease severity, as expected. The RPS values in this study were 71% and 76% after challenge with the IP and immersion methods, respectively. The specific *Fno* antibody titers and the RPS values obtained in this study correlated with those in previous studies that also used a whole-cell inactivated vaccine made from the highly virulent strain STIR-GUS-F2f7 isolated from red Nile tilapia mixed with the oil-based adjuvant Montanide^TM^ ISA 763 AVG [12,14]. In the study by Ramírez-Paredes et al. (2019), the strain STIR-GUS-F2f7 was used as the vaccine strain, as well as the challenge strain, in red Nile tilapia, wherein a high protection of 100% was obtained [14]. Later, Shahin et al. (2019) used the same vaccine, but challenged Nile tilapia with two more heterologous strains in addition to the homologous strain STIR-GUS-F2f7. An RPS of 82% was obtained against the challenge with the homologous strain, while the cross-protection obtained against the two heterologous strains amounted to RPS rates of 69.8% and 65.9% [12]. Moreover, our findings are also in agreement with those for the previous attenuated *Fno* vaccine, which demonstrated high protection (RPS 87.5%) after an immersion challenge [11]. However, the RPS in our vaccine revealed a minor difference in RPS values compared to previous studies. This could be due to the difference in the virulence of the bacteria tested (the mortality of our non-vaccinated fish was 91.65% at 21 days after the IP challenge), bacterial cultivation methods, and environmental conditions, and the origin and size of the fish tested. The fish tested in the current experiment weighed around 35 ± 5 g, but fish weights in the previous studies were 6.4 g and 15 g. Notably, the RPS obtained from the immersion challenge method in this study was higher than that from the IP injection method. The immersion challenge was considered to be more effective with regard to the lower mortality and higher RPS value. The fish challenged with the IP injection showed a higher mortality and lower RPS value because a direct injection would mean that the pathogens can bypass the first line of defense, including mucus and skin, and have direct access to the organs of predilection. But in the immersion challenge, bacteria need to penetrate the mucosal surfaces and skin before entering the system [48]. Therefore, the immersion challenge is more effective than the intraperitoneal challenge in reference to the RPS value. However, the IP route resulted in an acute onset of the disease and higher mortality than the immersion challenge method due to the pathogen quickly entering the blood vessels and internal organs. These findings also agree with the results of blood bacterial concentrations after challenge.

The blood bacterial concentrations at all time points—24, 48, 72, 120, and 168 h post-infection—were significantly lower than the bacterial concentration counts of the non-vaccinated control group. Consistent with these findings, in the spleen, head kidney, liver, and gills of vaccinated fish, both challenge methods showed a granuloma score of “level (**-**)” in more than 53.33% of fish. High granuloma scores of “level (+++)” in the spleen and kidney after the IP challenge of non-vaccinated fish were 86.67%, and for the fish challenged by immersion they were 86.67% and 66.67% in the spleen and kidney, respectively. Granulomatous lesions were prominent and numerous in the spleen and head kidney, which was similar to in previous studies [12,13,14,15]. The spleen and head kidney were selected as the organs for the assessment of granuloma scores because *Fno* prefers to multiply within macrophages, which are abundant in these lymphoid organs. [15,47]. Taken together, our results reveal the correlation between the survival rate, antibody titer, granuloma scores, and blood bacterial concentration in vaccinated fish. These data provide solid evidence that a *Fno*-specific antibody response is a useful protective immune response to *Fno* infection in fish as previously reported [12,13,14]. As *Fno* is a facultative intracellular organism, it can exist in an extracellular form, resulting in neutralization by antibodies and the subsequent control of infection. As a result, vaccinated fish in this study were able to reduce bacterial loads in the blood and show a marked reduction of granuloma scores after experimental infection.

## 5. Conclusions

These findings demonstrate the effectiveness, safety, convenience, and cost-effectiveness of a formalin-killed *Fno* vaccine against tilapia francisellosis. We were also successful in evaluating two different challenge methods, specifically the IP injection and immersion methods. Future studies are needed to evaluate the details of the protective effects, as well as the potential interplay between humoral and cell-mediated immune responses. In addition, further research on suitable adjuvants is also needed to develop an immersion vaccine. Furthermore, the use of other kinds of adjuvants or nanoparticles to formulate the vaccine is also a concern for improving the efficacy of an inactivated whole-cell *Fno* vaccine.

## Figures and Tables

**Table 1:** Primers used in qRT- PCR.

Genes	Primer	Sequence (5ʹ-3ʹ)
RPL23	otRPL23-F1	GTGTGTACGAAACAAGAACGAGCA
	otRPL23-R1	CACACACACACACACACACGAA
IL-1β	otIL-1βB-F1	AGTTGTGCTGTTTCTGGAGCAATAC
	otIL-1βB-R1	TCGCTCCATGTCTCTGTCAGTTAAA
TNFα	otTNFα-F1	GCTGGTCTCACTCATATGCACCTA
	otTNFα-R1	TGTCTTTTGGCAGACTGTACGGATA
CXCL8	otCXCL8-F1	CCTCCAAGAAACGGGCATAAATCC
	otCXCL8-R1	TCAGTCATGGCTCAGTGGTCAG
IL-17C	otIL17C-F1	CATCTTCGTACTGTTCATCGTGCC
	otIl17C-R1	TCCTTGTCGTTATAGCAGCGGAA

**Table 2:** The cumulative mortality and relative percent survival (RPS) value in vaccinated fish and the control group at 21 days and 60 days after challenge by the IP and immersion challenge methods, respectively.

Group	Fish Number	Mortality (%)	Average Mortality (%)	Average RPS (%)
	IP challenge			
Control 1	30	90	91.65	71
Control 2	30	93.39		
Vaccine 1	30	30	26.65	
Vaccine 2	30	23.3		
	Immersion challenge			
Control 1	30	60	61.67	76
Control 2	30	63.33		
Vaccine 1	30	16.67	15	
Vaccine 2	30	13.33		
